# Wound Healing Trajectories to Determine Pressure Ulcer Treatment Efficacy

**Published:** 2011-01-10

**Authors:** Wyatt G. Payne, Rajat Bhalla, Donald P. Hill, Yvonne N. Pierpont, Martin C. Robson

**Affiliations:** ^a^Institute for Tissue Regeneration, Repair, and Rehabilitation, Bay Pines VA Healthcare System, Bay Pines, Florida; ^b^Division of Plastic Surgery, University of South Florida, Tampa, Florida; ^c^Profil Institute for Clinical Research, Inc., Chula Vista, California

## Abstract

**Background:** Wound healing trajectories (percent healing vs time) provide a dynamic picture of the decrease in wound burden over the entire continuum of the healing process. Trajectories can be robustly compared using survival statistics methodology. Improvement in healing can be determined by shifting the curve from “impaired” healing toward “ideal” healing. Although this concept of shifting the curve “to the left” has been demonstrated in acute incisional healing depicted by the gain in tensile strength, and in other chronic wounds, it has not been utilized for chronic pressure ulcers. **Methods:** Wound healing trajectories were constructed for 211 patients enrolled in 8 separate randomized clinical trials for grade III and IV pressure ulcers. Trajectories were constructed for patients achieving ≥90% or more healing within 112 days and those who achieved less than <90% wound closure. Kaplan-Meier curves were constructed for all patients receiving an experimental treatment and for those receiving placebo vehicles. **Results:** Different trajectories were achieved for the faster healing patients. Eighty-one percent of patients reached 90% healing within 112 days; 80% of those in treatment groups and 85% of those in placebo groups. Linear regression suggested that all patients entered into the clinical trials would achieve 90% healing by 18 weeks. Only 17% of the patients achieved total healing (100% wound closure) within the 112-day study period. Linear regression suggested that it would take 110 weeks to achieve total healing in all patients. **Conclusion:** Wound healing trajectories provide a more complete description of treatment efficacy than do fixed endpoints, such as the number of patients achieving 100% closure at one defined time point. Since more successful healers have different trajectories than less successful healers, shifting the trajectory to the left from “impaired” toward “ideal” healing may provide a better endpoint to determine treatment efficacy.

Wound repair processes depend on the interaction of many time-dependent components.^1^ A wound healing trajectory allows one to evaluate the outcome of healing versus time. Knowing time for healing is vital, as increased time correlates with greater rates of infection and scarring. The wound healing trajectory integrates the many time-dependent processes that are part of the healing process and is affected by systemic and local deterrents to healing. These trajectories clearly display increased healing (reflected by a left shift of the curve) or “impaired” healing (reflected by a right shift of the curve). Actual wound healing is different from “ideal” wound healing due to deterrents to healing, which shift the trajectory to the right toward “impaired” healing. The goal for an agent attempting to accelerate the healing process is to shift the curve to the left, toward the “ideal.”^1^

Because healing of open wounds follows an exponential curve, wound healing trajectories (percent wound closure vs time) have been used to describe chronic wound healing.^1^^-^3 Although wound healing trajectories were initially described for acute wounds, they can also be used to evaluate the healing of chronic wounds, such as diabetic ulcers, pressure ulcers, and venous stasis ulcers. The trajectory curve, similar to the Gompertz growth curve for biological systems, is sigmoid-shaped, with time on the x-axis and percentage of wound closure on the y-axis.^4^^-^6,7 This exponential healing process is based on the equation expressed by DuNouy,^8^ with the rate of change of wound area decreasing as the residual wound area approaches total closure.

Trajectories allow examination of the entire continuity of wound healing, at any time point rather than at several distinct points. Although a number of models have been proposed to describe wound healing (both linear and nonlinear), we have utilized the wound healing trajectory approach proposed by Hokanson et al,^9^ which leaves the raw data for wound area free of mathematical transformation.^4^,^8^ Using this mathematical model, we have been able to approximate the actual wound closure measurements and utilize endpoint analysis similar to that used in failure-time studies. This analytical strategy has been applied successfully to large groups of experimental wound healing studies.^4^ In this method of analysis, a wound healing trajectory is created on the basis of a plot of percentage of wound closure versus time of wound treatment and can then be used to predict healing and determine the efficacy of a therapeutic agent.

With this wound healing trajectory plot, the time required to achieve a certain percentage of healing can be measured, using survival study methods as described by Kaplan and Meier.^9^,^10^,^15^ Utilizing statistical analysis methods used for failure-time or survival analysis, the distributions of fractional closure times can be examined for statistical differences.^11^ The usefulness of wound healing trajectories as predictors of efficacy of therapeutic agents for diabetic foot ulcers and venous stasis ulcers has been previously demonstrated and validated.^4^,^5^ Wound healing trajectories as outcome measures for pressure ulcer treatment appear to be a useful tool for predicting healing times and can be utilized to assess treatment regimens.

## METHODS

Wound healing trajectories were constructed from 211 patients enrolled in 8 randomized clinical trials for grade III and IV pressure ulcers.^6^ All studies were approved by institutional review board for human studies. Patients were treated between 28 and 112 days, depending on the specific protocol. The inclusion/exclusion criteria, standards of care, and care providers were similar for all patients. Pressure ulcer size was quantified utilizing jeltrate molds; serial molds were used to describe healing trajectories during the course of the treatment protocols. Healing fractions (mold size on day *X*/mold size on day 0) were used to describe the course of healing over time. Healing fractions provided the data for both the healing trajectories and Kaplan-Meier survival analyses. Trajectories were constructed for patients achieving ≥90% (faster) and < 90% (slower) healing within 112 days. Kaplan-Meier curves were constructed for all patients receiving an experimental treatment and for those receiving placebo vehicles.

## STATISTICAL METHODOLOGY

Survival analyses were performed with the Kaplan-Meier method, using log-rank and Wilcoxon statistics to test between groups. Calculations and graphs were constructed utilizing SigmaStat and SigmaPlot software (SPSS, Chicago, Ill), and also JMP software (SAS, Carey, NC).

## RESULTS

**Figure 1:**
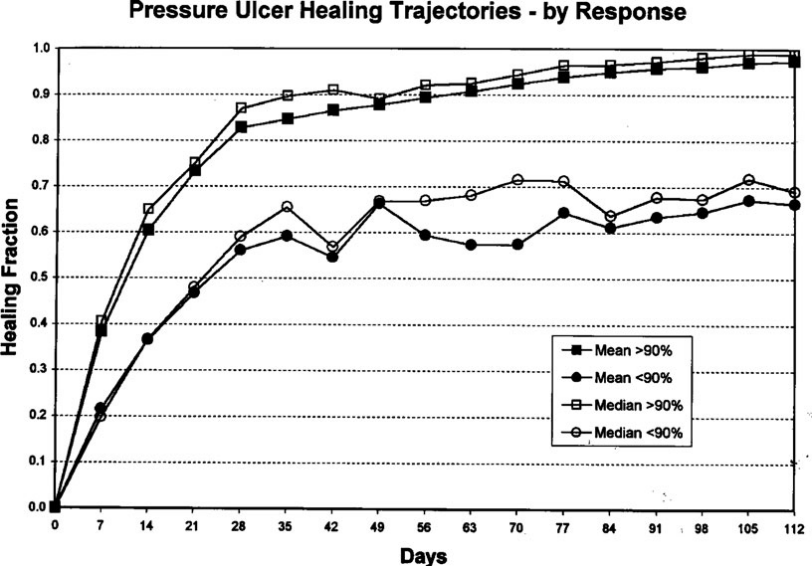
Curves depicting mean and median values for healing fraction over 112 days. Squares indicate patients who achieved ≥90% wound closure; circles indicate patients who achieved less than <90% wound closure. Healing fraction refers to percentage of initial wound, calculated as wound mold size on day *X*/wound mold size on day 0.

**Figure 2:**
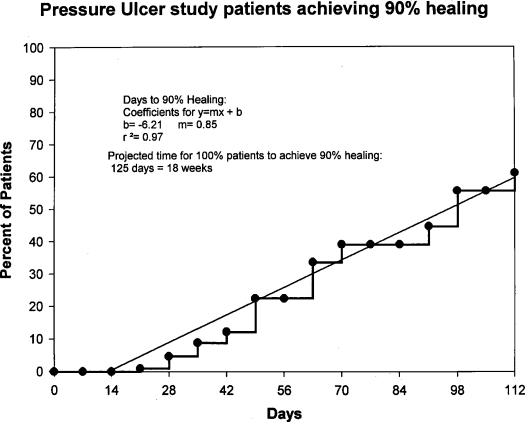
Kaplan-Meier healing curve depicting the percentage of patients achieving 90% wound closure. Linear regression indicates that all patients would achieve 90% healing by 18 weeks.

**Figure 3:**
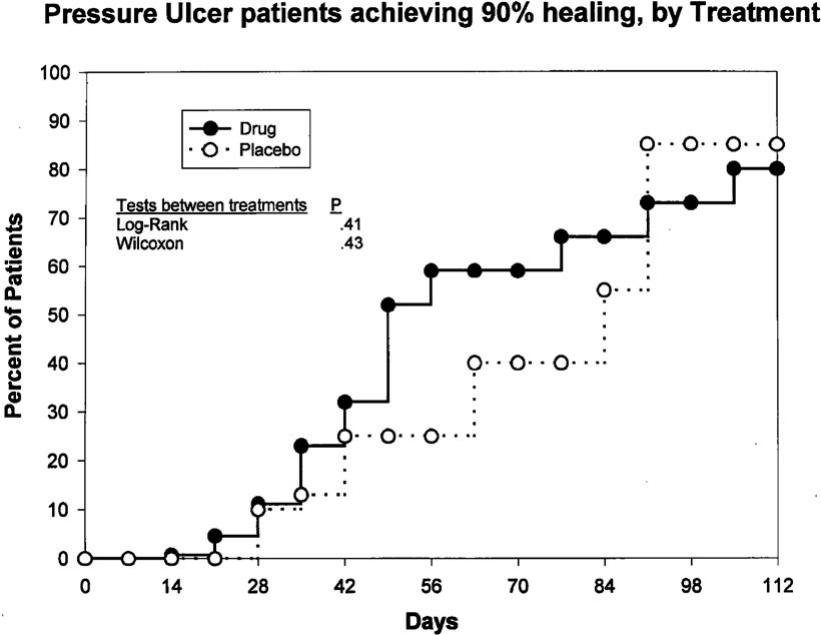
Kaplan-Meier healing curves depicting percentage of patients achieving 90% wound closure, by treatment. Eighty percent of those in all treatment groups combined and 85% of those in all placebo groups combined achieved 90% healing within 112 days; these differences were not significant (Wilcoxon, *P* = .43; and log rank test, *P* = .41). Significant differences were seen among treatment and placebo groups within individual studies (data not shown).

**Figure 4:**
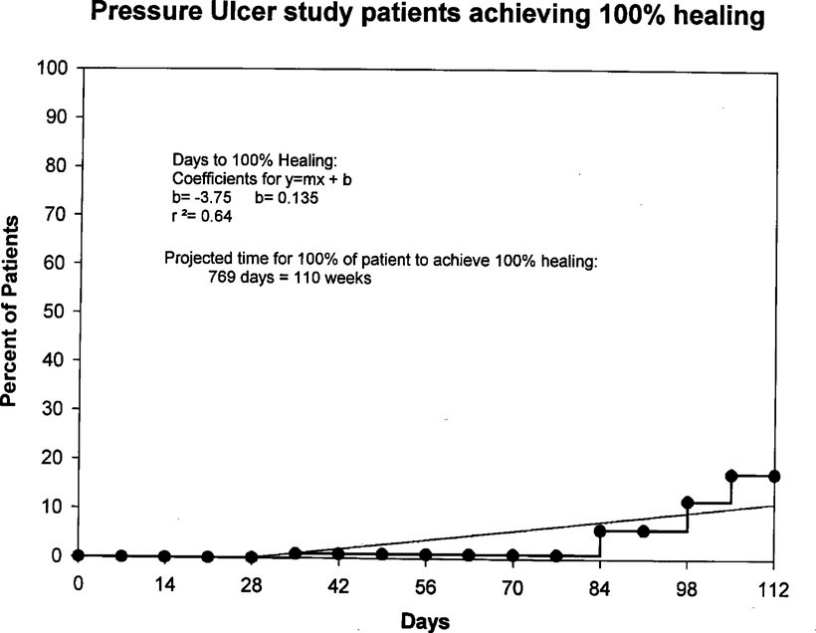
Kaplan-Meier healing curve depicting percentage of patients achieving 100% wound closure. Seventeen percent of patients achieved total healing within 112 days. Linear regression of survival analysis data suggests that it would take almost 110 weeks to achieve total healing in all patients.

Figure 1 depicts the markedly different trajectories for patients in the faster versus slower healing groups. Survival analysis suggests that 81% percent of all patients reached 90% healing within 112 days. A linear regression (Fig 2) of these data indicates that all patients in these clinical trials would achieve 90% healing by 18 weeks. Survival analysis by treatment groups (Fig 3) suggests that 80% of those in all treatment groups combined and 85% of those in all placebo groups combined achieved 90% healing within 112 days; these differences were not significant (Wilcoxon, *P* = .43; and log rank test, *P* = .41). Significant differences were seen among treatment and placebo groups within individual studies (data not shown). Only 17% of the patients achieved total healing (100% wound closure) within the study period of 112 days (Fig 4). Linear regression of survival analysis data suggested that it would take almost 110 weeks to achieve total healing in all patients.

## DISCUSSION

The goal of most clinical wound healing research is to discover and/or study products and processes that might accelerate wound healing.^12^ One limiting factor in clinical trials testing therapeutic agents has been trial design, including the methodology and determination of endpoints.^4^ To evaluate treatment efficacy, outcome measures or clinical endpoints are necessary, and a number of studies have suggested various outcome measures for the healing of open chronic wounds.^4^ According to the US Food and Drug Administration, the only acceptable treatment outcome for wounds is total wound healing (100% closure).^13^ In the study trials, only 17% of patients achieved total healing within the allotted study period, 112 days (Fig 4). The data suggest that all patients receiving the proper level of wound care will achieve 100% wound closure by 110 weeks. Therefore, a clinical trial would have to last at least 110 weeks, an impractical and expensive endeavor, to allow for the best determination of this efficacy endpoint (100% closure). Clearly, the time to 100% healing in all patients places unreasonable time constraints on patient participation in treatment protocols. The tremendous costs of such a large clinical trial point to a great need for intermediate endpoints that can serve as efficacy endpoints, hence a focus on wound healing trajectories.

The wound healing trajectory has been shown to be a valid and useful representation of the exponential course of the healing of wounds, with the rate of change of wound area progressively decreasing as the residual wound area approaches total closure.^4^,^14^ Wound healing trajectories describe the entire continuum of the wound healing process, as opposed to statistical analyses like the *t* test that are performed on a single point.^8^ These trajectories provide better insight into treatment efficacy than do fixed endpoints, that is, the number of patients achieving 100% closure at one predefined time point.^15^ With these trajectories, the time required to reach a certain percentage of healing can be calculated. These distributions of times to an event (90% wound closure) can then undergo standard statistical failure-time analysis and can be just as rigorous as total (100%) ulcer healing statistics.

Trajectories can be utilized for both acute and chronic wounds alike, but in this study, we have focused on chronic wounds and specifically examined the healing trajectories of pressure ulcers. Although partial healing is not recognized as an acceptable wound healing endpoint by the Food and Drug Administration, the use of wound healing trajectories to determine time to a certain level of partial healing can be considered an efficacy outcome measure as previously described.^4^,^5^,^11^ Venous stasis ulcers and diabetic foot ulcers show trajectory outcomes from independent clinical trials that closely mimic each other. In addition, the trajectories of patients achieving 100% closure in a certain time period were very different from the trajectories of patients not achieving total healing in that same time period.

The results of this current study show that more successful healers have different trajectories than less successful healers. Therefore, a shift of the trajectory to the left from “impaired” toward “ideal” healing may provide a better endpoint to determine treatment efficacy.^1^ The concept of a trajectory shift of patients with less than 100% closure in a defined period to the trajectory of patients attaining total ulcer closure could be used to determine new therapeutic agent efficacy. Our goal in this study was to point out the predictive value of wound healing trajectories for pressure ulcers. Ideally, shorter clinical trials can be constructed relying on specific shifts of the wound healing trajectories from impaired healing toward an ideal endpoint.^4^,^5^

## CONCLUSION

Wound healing trajectories appear to be a useful tool for evaluation of time to healing, especially with the utilization of clinical data. Wound healing trajectories have been shown to be of utility in the evaluation of acute wounds and of chronic wounds including venous stasis ulcers and diabetic foot ulcers. This current study validates wound trajectory utilization for pressure ulcers. Using wound healing trajectories may allow for more efficient clinical trial protocol design in future wound healing trials.

## Acknowledgments

This material is the result of the work supported with resources and the use of facilities of the Bay Pines V.A. Healthcare System. The contents of this work do not represent the views of the Department of Veterans Affairs or the US government.
